# Effects of nitrogen fertilization and bioenergy crop type on topsoil organic carbon and total Nitrogen contents in middle Tennessee USA

**DOI:** 10.1371/journal.pone.0230688

**Published:** 2020-03-30

**Authors:** Jianwei Li, Siyang Jian, Chad S. Lane, YueHan Lu, Xiaorui He, Gangsheng Wang, Melanie A. Mayes, Kudjo E. Dzantor, Dafeng Hui

**Affiliations:** 1 Department of Agricultural and Environmental Sciences, Tennessee State University, Nashville, TN, United States of America; 2 Department of Earth and Ocean Sciences, University of North Carolina Wilmington, Wilmington, NC, United States of America; 3 Department of Geological Sciences, University of Alabama, Tuscaloosa, Alabama, United States of America; 4 Institute for Environmental Genomics and Department of Microbiology & Plant Biology, University of Oklahoma, Norman, Oklahoma, United States of America; 5 Oak Ridge National Laboratory, Climate Change Science Institute and Environmental Sciences Division, Oak Ridge, Tennessee, United States of America; 6 Department of Biological Sciences, Tennessee State University, Nashville TN, United States of America; Sichuan Agricultural University, CHINA

## Abstract

Nitrogen (N) fertilization affects bioenergy crop growth and productivity and consequently carbon (C) and N contents in soil, it however remains unclear whether N fertilization and crop type individually or interactively influence soil organic carbon (SOC) and total N (TN). In a three-year long fertilization experiment in switchgrass (SG: *Panicum virgatum* L.) and gamagrass (GG: *Tripsacum dactyloides* L.) croplands in Middle Tennessee USA, soil samples (0–15cm) were collected in plots with no N input (NN), low N input (LN: 84 kg N ha^-1^ yr^-1^ in urea) and high N input (HN: 168 kg N ha^-1^ yr^-1^ in urea). Besides SOC and TN, the aboveground plant biomass was also quantified. In addition to a summary of published root morphology data based on a separated mesocosm experiment, the root leachable dissolved organic matter (DOM) of both crops was also measured using archived samples. Results showed no significant interaction of N fertilization and crop type on SOC, TN or plant aboveground biomass (ABG). Relative to NN, HN (not LN) significantly increased SOC and TN in both crops. Though SG showed a 15–68% significantly higher ABG than GG, GG showed a 9.3–12% significantly higher SOC and TN than SG. The positive linear relationships of SOC or TN with ABG were identified for SG. However, GG showed structurally more complex and less readily decomposed root DOM, a larger root volume, total root length and surface area than SG. Collectively, these suggested that intensive N fertilization could increase C and N stocks in bioenergy cropland soils but these effects may be more likely mediated by the aboveground biomass in SG and root chemistry and morphology in GG. Future studies are expected to examine the root characteristics in different bioenergy croplands under the field fertilization experiment.

## Introduction

Perennial switchgrass (SG: *Panicum virgatum* L) and gamagrass (GG: *Tripsacum dactyloides* L) are two important bioenergy crops that are common alternative energy sources for sustainable replacement of fossil fuels [1–3]. Added together with other cellulosic biofuel crops, these dedicated energy crops will contribute to more than 30% of biofuel plant biomass in the coming decades [2, 4]. N fertilizers generally increase bioenergy crop yields [5, 6], but many studies report highly varied magnitudes and signals of soil C and N contents in response to N fertilization [7–10]. Few studies have compared the root traits in different bioenergy crops and no study has investigated the role of root traits in mediating bioenergy crop and soil responses to N fertilization. Elucidating the effects of fertilization on plant and soil C and N dynamics will provide fundamental knowledge needed to develop effective strategies to improve soil quality, C sequestration, agricultural productivity, and climate change adaptation [11–13].

Past studies showed no consistent pattern of N fertilization effect on SOC and TN contents. N fertilizations can enhance SOC and TN contents by 9–45% in SG croplands [8, 14–20]. Under an intensive N fertilization regime (e.g., 180 kg N ha^-1^ yr^-1^), SOC enhancement is reported due to C accretion from elevated root C input and reduced input of particulate organic C [7, 21]. In another study, both inorganic and manure N fertilizations can improve SOC sequestration capacity in SG croplands [15], which is associated with elevated shoot and root biomass [22]. In other studies, N fertilizations, however, show no significant effects on SOC pools in a soil profile (0–100cm) at a fertilization rate between 0 and 220 kg N ha^-1^ yr^-1^ [17, 23, 24]. A similar conclusion was reached in a fertilization experiment of short-term period of 2–3 years after SG establishment [20]. On the other hand, little change in soil TN under fertilization can be derived based on the N budget for annual SG production, which was closely balanced with 6.3 g N m^-2^ removed by harvest of aboveground biomass and 6.7 g N m^-2^ supplied by fertilization [25]. Though not common, N fertilizations can also diminish SOC and TN stocks, and this effect is particularly evident in the stable, mineral-associated C and N pools at depths greater than 15 cm [8]. To our best knowledge, no studies have reported soil C and N storages in response to N fertilization in GG croplands. It also remained unknown whether there was significant interaction of N fertilization and bioenergy crop type on SOC and TN stocks.

The large variations of SOC and TN in response to N fertilization are typically attributed to the perennial nature of bioenergy crops and their deep-rooted growth form [18]. Relative to SG, GG is reported to possess larger roots, higher root biomass and volume [26], total root length and surface area [27]. This contrasting root morphology may favor accrual of SOC and TN [28, 29]. Besides the root morphology, plant litter and root chemistry of bioenergy crops may also influence SOC and TN changes under N fertilization. Gil and Fick [30] identified a higher plant biomass C:N ratio for GG than other crops, which correlated strongly with lower net N mineralization and losses thus favoring C and N sequestrations [31]. The *in situ* root chemistry (C:N) of bioenergy crops is rarely quantified but our recent work found that both plant shoot and root C:N differed largely between SG and GG based on a mesocosm experiment. These studies focused on plant traits, but provided little information of C and N changes in soil simultaneously so that understanding the interaction of soil and bioenergy crop is hindered.

Knowing the abundance of humic-like or protein-like compounds will offer information of chemical recalcitrance [32, 33], but this analysis has not been conducted for root of bioenergy crops. A study revealed that the structural-tissue-dominated slow turnover root C pool concentrated at surface soil horizons in a prairie [34] and this indicated a strong linkage of root chemistry with soil C and N storage. However, no study has simultaneously quantified soil response and root traits (e.g., morphology and chemistry) in order to explore how root traits likely moderate soil responses in bioenergy crops. Using a bioenergy crop field experiment in Middle Tennessee, we investigate the effects of N fertilization on the elemental characteristics of plant and soil C and N in two bioenergy croplands (SG and GG). N fertilization represents the primary management practice in our research plots with no tillage, plowing, or minor mechanical disturbance applied during the experimental period.

Given the different nature of SG and GG roots (i.e., chemistry and morphology), we first hypothesize that there is a significant interaction of N fertilization and bioenergy crop type on SOC, TN and plant aboveground biomass such that N fertilization-enhanced SOC, TN and plant aboveground biomass was more pronounced in GG than SG. Alternatively, there is only significant N fertilization effect. In that scenario, we establish the second hypothesize that the N fertilization effect will be significant only under a high fertilization rate because the low fertilization effect can be masked due to large variations in field measurements. Based on a mesocosm experiment examining the two bioenergy crop (SG and GG) seedlings’ characteristics, we set up the third hypothesize that the root leachable dissolved organic matter (DOM) is more structurally complex and less easily decomposed for GG than SG because GG root is larger based on the published data synthesis of root morphology in the same mesocosm study. Although we lack fertilization treatment in the mesocosm study, the root morphology and chemistry of the two bioenergy crops are compared and linked to SOC and TN changes in response to N fertilization.

## Materials and methods

### Site description, soil and plant sample collections

Initially established in 2011, the bioenergy crop field fertilization experiment is located at the Tennessee State University (TSU) Main Campus Agriculture Research and Education Center (AREC) in Nashville, TN, USA (Lat. 36.12° N, Long. 36.98° W, elevation 127.6 m above sea level). Prior to the establishment of switchgrass and gamagrass croplands, the land use type was the mowed grassland for several decades. No fertilizers were applied during the prior land use. Climate in the region is a warm humid temperate climate with an average annual temperature of 15.1°C, and total annual precipitation of 1200 mm [35]. The crop type and N fertilization treatments were included in a randomized block design [27, 36]. The two crop types were *Alamo* SG (*Panicum virgatum* L.) and GG (*Tripsacum dactyloides* L.). The three N levels included no N fertilizer input (NN), low N fertilizer input (LN: 84 kg N ha^-1^ yr^-1^ as urea), and high N fertilizer input (HN: 168 kg N ha^-1^ yr^-1^ as urea), and each treatment had four replicated plots with a dimension of 3 m × 6 m. The low N fertilization rate was determined as the optimum N rate to maximize cellulosic ethanol production in established northern latitude grasslands [37]. The high N rate doubled the low rate in order to create appreciable gap and detectable effect between the two levels. The fertilizer was manually applied in June or July each year after cutting the grass. The soil series for the plots is Armour silt loam soil (fine-silty, mixed, thermic Ultic *Hapludalfs*) with acidic soil pH (i.e., 5.97) and intermediate organic matter content of 2.4% [36, 38].

In the fertilization experiment, soil samples (0–15 cm) were collected from 12 plots (2 crops × 3 N inputs × 2 replicated plots) on June 6, 2015. Within each plot, 24 cores were randomly collected using a spatially explicit sampling design [39] and a total of 288 soil cores were obtained in 12 plots. This soil sampling design has been used to quantify the spatial heterogeneity of soil microbial biomass, SOC and TN in the same experiment [36, 40], and in a former study [41]. The soil samples were transported to the TSU lab in a cooler filled with ice packs and were then subsequently stored at 4°C until analysis. Visible roots and rocks were removed from the samples, and soil samples were then passed through a 2 mm soil sieve. SOC and TN concentrations were analyzed using a Costech 4010 elemental analyzer (*Costech analytical technologies Inc*., Valencia, CA, USA). Although 24 samples were collected and analyzed in each replicated plot (i.e., used to map soil C and N spatial heterogeneity in another manuscript), the mean values of SOC, TN, and C:N were obtained in each plot and applied in the ANOVA test in order to avoid the artificial effect of pseudo-replication [42]. This generated 12 samples (3 fertilization × 2 crop × 2 replicate).

Harvesting of SG and GG aboveground (ABG) biomass was conducted twice in four replicated plots under each of three fertilization treatments during June to October in 2014 and 2015. This resulted in 24 samples in each year (2 crops × 3 N inputs × 4 replicated plots). At each harvest, plants were cut 7 inches above the ground using a Carter Mfg. Co plot harvester with flail cutters and mounted module capable collecting biomass fresh weights in the field. In each plot, subsamples of fresh biomass per unit area were dried to constant weight at 70°C using an Oven King industrial capacity dryer (Washington Industrial Corp. Seattle, WA, USA) to determine dry biomass. The unit of biomass was expressed as Mg ha^-1^. To analyze biomass C and N concentrations, subsamples of dry biomass in 2014 and 2015 were selected and one composited sample was obtained by equal weight of sample for each crop under each fertilization treatment (i.e., NN, LN, and HN). This generated 6 samples (3 fertilization × 2 crop). Plant samples were analyzed for C and N concentrations using a Costech 4010 elemental analyzer (*Costech analytical technologies Inc*., Valencia, CA, USA).

### The mesocosm experiment, root sample collection and analysis

The root materials of SG and GG were obtained from the historical archived samples collected from a mesocosm experiment [27]. Briefly, the experiment was conducted in the greenhouse of the TSU campus farm in 2015 when the SG and GG seedlings were planted in tree pots for three months. Before planting, seeds were germinated in potting mix (FafardH #2 mix). At the 3– to 4–leaf stage, seedlings were transplanted into 15–cm wide x 41–cm high tree pots (Stuewe and Sons, https://www.stuewe.com/products/treepots.php), each containing 6 kg of soil. The pot pH was set at 6.5, which is similar to the acidic soil pH in the field fertilization experiment (i.e., 5.97). Each treatment was replicated eight times. After 3 months, root samples were cut from below the soil surface and rinsed thoroughly with DI water. Root samples were dried in 70°C to constant weight. The root traits including surface area, length, and biomass productivity were compared between SG and GG and the results have been published formerly [27].

For this study, eight replicated root samples for both crops were selected to analyze the abundance and components of dissolved organic matter (DOM) leached from root samples. This generated 16 samples. This analysis was conducted at the Molecular Eco-Geochemistry laboratory of University of Alabama. For DOM leaching, root powders were mixed with carbon-free ultrapure water at a ratio of around 1:8 by mass for most samples. If slurry-like mixture appeared at this ratio, extra water was added until a clear liquid layer appeared. The mixtures were constantly agitated for 42 hours on an orbital shaker at 300 rpm, followed by centrifugation at 4,000 rpm for 20 minutes. The upper liquid layer was carefully transferred to a new vial using a pipette and the leachable DOM in these samples was further analyzed for absorbance and fluorescence properties (i.e., Excitation-Emission Matrix coupled with Parallel Factor Analysis), following the analytical methods described in detail in former publications [33, 43].

Here we briefly described the procedures on how to conduct the DOM absorbance and fluorescence property analysis. The absorbance of DOM was analyzed using a UV-1800 Shimadzu spectrophotometer, and the spectra from the wavelength of 190 to 670 nm at a 1 nm interval were collected. Three-dimensional fluorescence excitation-emission matrices (EEM) were analyzed using a Horiba Jobin-Yvon Fluoromax-3 spectrofluorometer, with the reading collected at excitation wavelengths from 240 to 500 nm at 5 nm intervals and emission wavelengths from 280 to 538 nm at 3 nm intervals. The EEM spectra were corrected for blanks, the inner filter effect, and the manufacturer’s correction factors and subsequently normalized relative to the area under the water Raman peak [44]. A series of optical indices are calculated to interpret DOM source and compositional characteristics: 1) slope ratio (S_R_) of absorbance of 275–295nm over 350–400nm, which is negatively correlated with DOM molecular weight [45, 46]; 2) the ratio of E2: E3 (ratio of absorbance at 250 to 365 nm), which decreases as DOM molecular size increases [47]; 3) fluorescence index (FI), for which lower values are thought to represent larger, structurally more complex compounds usually produced from terrestrial plant decay [48]; 4) the ratio of C to T, which indicates the relative amount of humic-like (recalcitrant) versus protein-like (labile) compounds in a sample [32]; and 5) humification index (HIX), for which greater values correspond to an increasing degree of humification [49, 50].

The parallel factor analysis (PARAFAC) was conducted in MATLAB using the DOMFluor toolbox described in detail by [51], and the final model was validated using the split-half analysis [52]. Based on fluorescence excitation-emission matrix [32, 53], the PAFRAC model was used to identify three components–C1 and C2 representing protein-like DOM and C3 representing humic-like DOM (S1 Table). Given that tyrosine-like DOM is found to be the first component to decrease in leaf leachate during the senescence [54], a high percent tyrosine-like DOM and low tryptophan-like DOM in a sample indicate more labile SOM to microbial degradation.

### Statistical analysis

Two-way analysis of variance (ANOVA) was used to test the main and interactive effects of N fertilization and crop type on SOC, TN, and C: N, and plant ABG biomass in 2014 and 2015. Tukey HSD *Post hoc* tests were conducted to compare the means when a main or interactive effect is significant. To conduct the ANOVA, the original data was log transformed if it violated equal variance assumption. The regression plots between SOC, TN and plant ABG biomass were also obtained for SG and GG. One-way ANOVA was used to examine the effect of crop type on the indices of DOM leached from root (S_R_, E2: E3, FI, and HIX; CT, Tyrosine-like DOM, Tryptophan-like DOM and Humic DOM). These analyses were conducted using R [55]. The significance level was set at *P* < 0.1. This threshold *p-value* was selected to accommodate the likely high variability of initial soil C and N contents at the beginning of the experiment.

## Results

### SOC, TN, C: N, and ABG biomass under fertilization in SG and GG

There was no significant interactive effect of fertilization and crop type on SOC, or TN (*P* > 0.1; Table 1). There were significant effects of fertilization and crop type on SOC and TN (*P* < 0.1; Table 1). Post hoc tests indicated that relative to NN, LN insignificantly increased SOC and TN by 2.5% and 2.8%, and HN significantly increased SOC and TN by 15% and 17%, respectively (Table 2). Relative to SG, GG showed a significantly 9.3% higher SOC and 12% higher TN (Table 2). Last, no significant fertilization or crop type effect on C: N was detected, but their interaction effect is significant (*P* < 0.1; Table 1).

**Table 1:** *p*-values of two-way ANOVA statistical tests on the main and interactive effects of N fertilization and crop type on SOC, TN and C: N as well as aboveground plant biomass (ABG) in 2014 and 2015 under three fertilization treatments in SG and GG croplands at the fertilization experiment in TSU AERC in Nashville, TN, USA.

Variable	N fertilization	Crop	Crop×N fertilization
SOC	**0.072**	**0.082**	0.878
TN	**0.057**	**0.049**	0.429
C: N	0.401	0.163	**0.059**
ABG (2014)	0.434	**0.097**	0.821
ABG (2015)	0.144	**0.025**	0.463

Bold numbers denote P < 0.1.

**Table 2:** Mean (±SE) SOC and TN concentrations (%), and C: N as well as their respective coefficients of variance (CV, %) under three fertilization treatments in SG and GG croplands at the fertilization experiment in TSU AERC in Nashville, TN, USA.

Crop	N Fertilization	SOC		TN		C: N	
		Mean±SE	CV	Mean±SE	CV	Mean±SE	CV
		% %		% %		%	
SG	NN	1.48±0.005^a^	0.45	0.13±0.002^a^	1.86	11.05±0.09^a^	1.13
	LN	1.56±0.09^a^	7.91	0.15±0.01^a^	11.23	10.57±0.27^a^	3.61
	HN	1.72±0.03^b^	2.32	0.17±0.0003^b^	0.26	10.27±0.18^a^	2.45
GG	NN	1.66±0.11^a^	9.30	0.16±0.007^a^	6.08	10.10±0.23^a^	3.15
	LN	1.66±0.14^a^	11.52	0.16±0.02^a^	14.04	10.62±0.22^a^	2.95
	HN	1.89±0.07^b^	5.48	0.18±0.004^b^	3.51	10.40±0.13^a^	1.73

SG: switchgrass; GG: gamagrass; NN: No N input; LN: Low N fertilizer input (84 kg N ha^-1^ yr^-1^)

HN: High N fertilizer input (168 kg N ha^-1^ yr^-1^)

**Fig 1:**
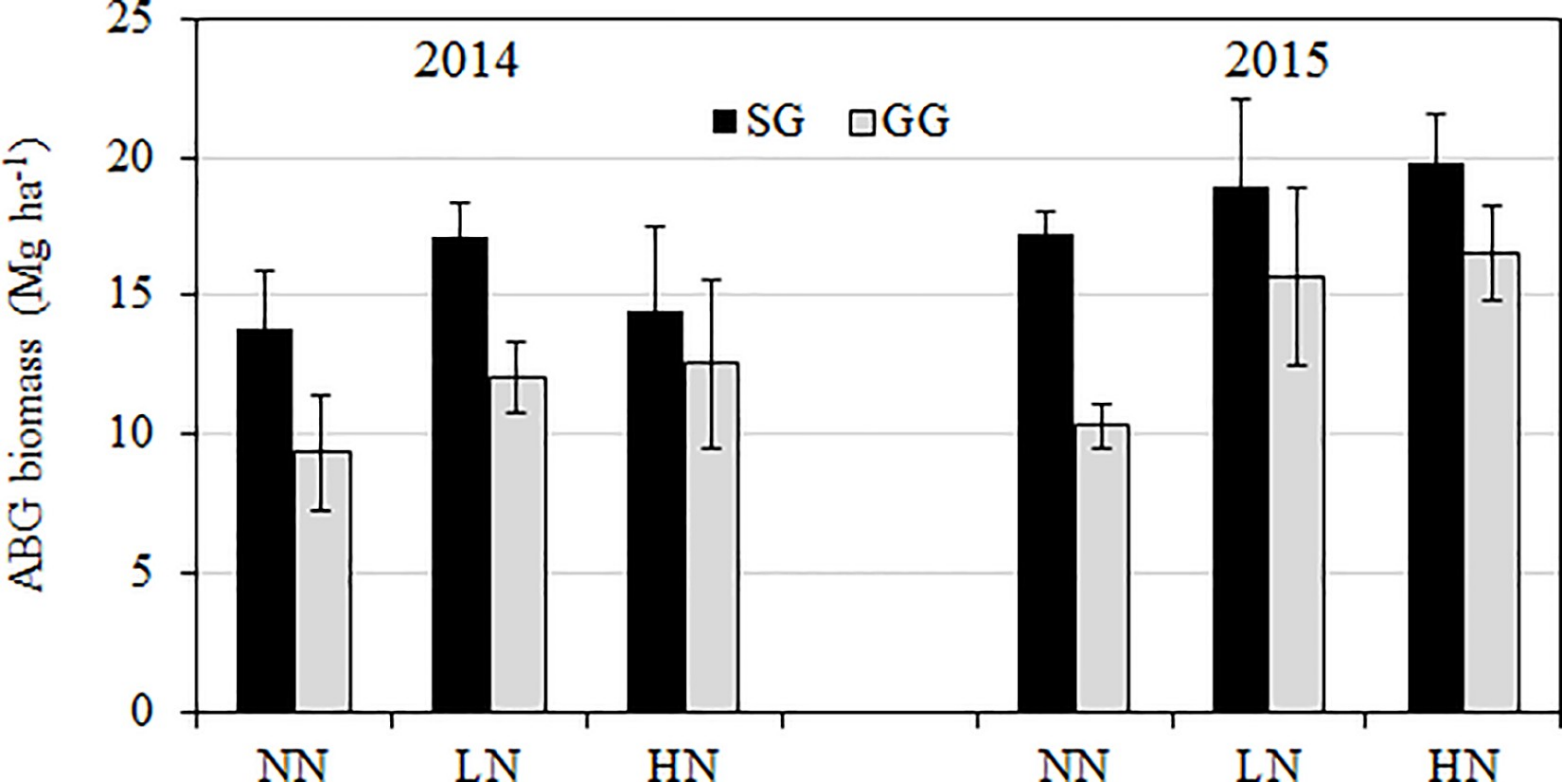
Mean (±SE) plant ABG biomass (Mg ha^-1^) under three fertilization treatments (e.g, NN, LN and HN) in SG and GG croplands in 2014 and 2015. There was only significant crop type effect on ABG biomass in each collection year (Table 1). Each bar represents a mean value of four replicates (n = 4). NN: no N input; LN: low N input (84 kg N ha^-1^ in urea); HN: high N input (168 kg N ha^-1^ in urea); ABG: aboveground.

**Fig 2:**
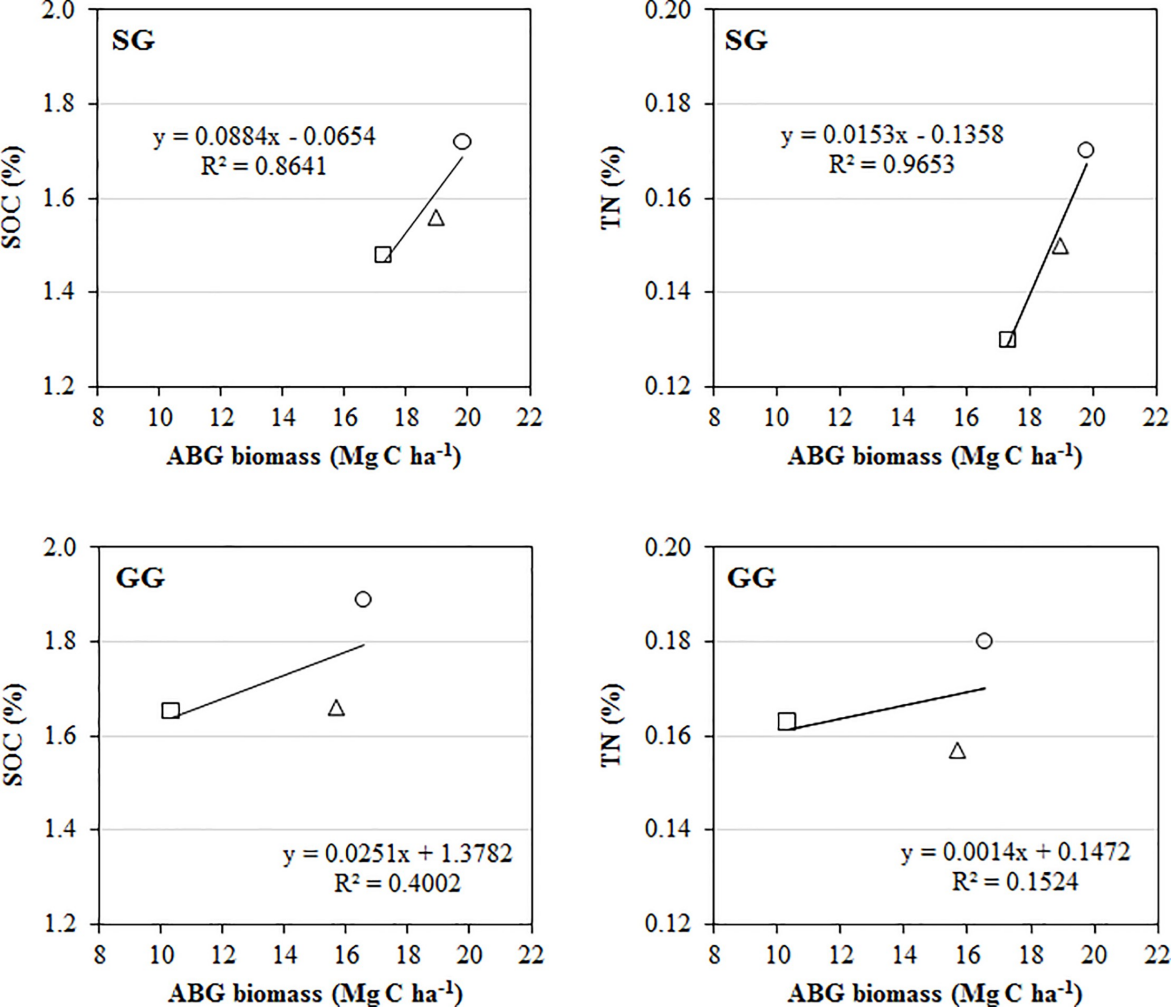
Regression plots of mean SOC, TN and plant ABG biomass under NN (□ square), LN (Δ triangle) and HN (○ circle) in SG and GG croplands. Plant ABG biomass was referred to the collection in 2015 only. NN: no N input; LN: low N input (84 kg N ha^-1^ in urea); HN: high N input (168 kg N ha^-1^ in urea); ABG: aboveground.

There was no significant fertilization effect or interaction of fertilization and crop type on ABG, but there was significant effect of crop type on ABG in both collections in 2014 and 2015 (*P* < 0.1; Table 1). Post hoc tests indicated a significantly higher ABG by 15–68% in SG than GG (Fig 1). The regression plots of SOC, TN and ABG biomass showed stronger linear relationship of SOC or TN with ABG for SG (R^2^ > 0.86) than those for GG (R^2^ < 0.41) (Fig 2).

### DOM chemistry of SG and GG roots

**Fig 3:**
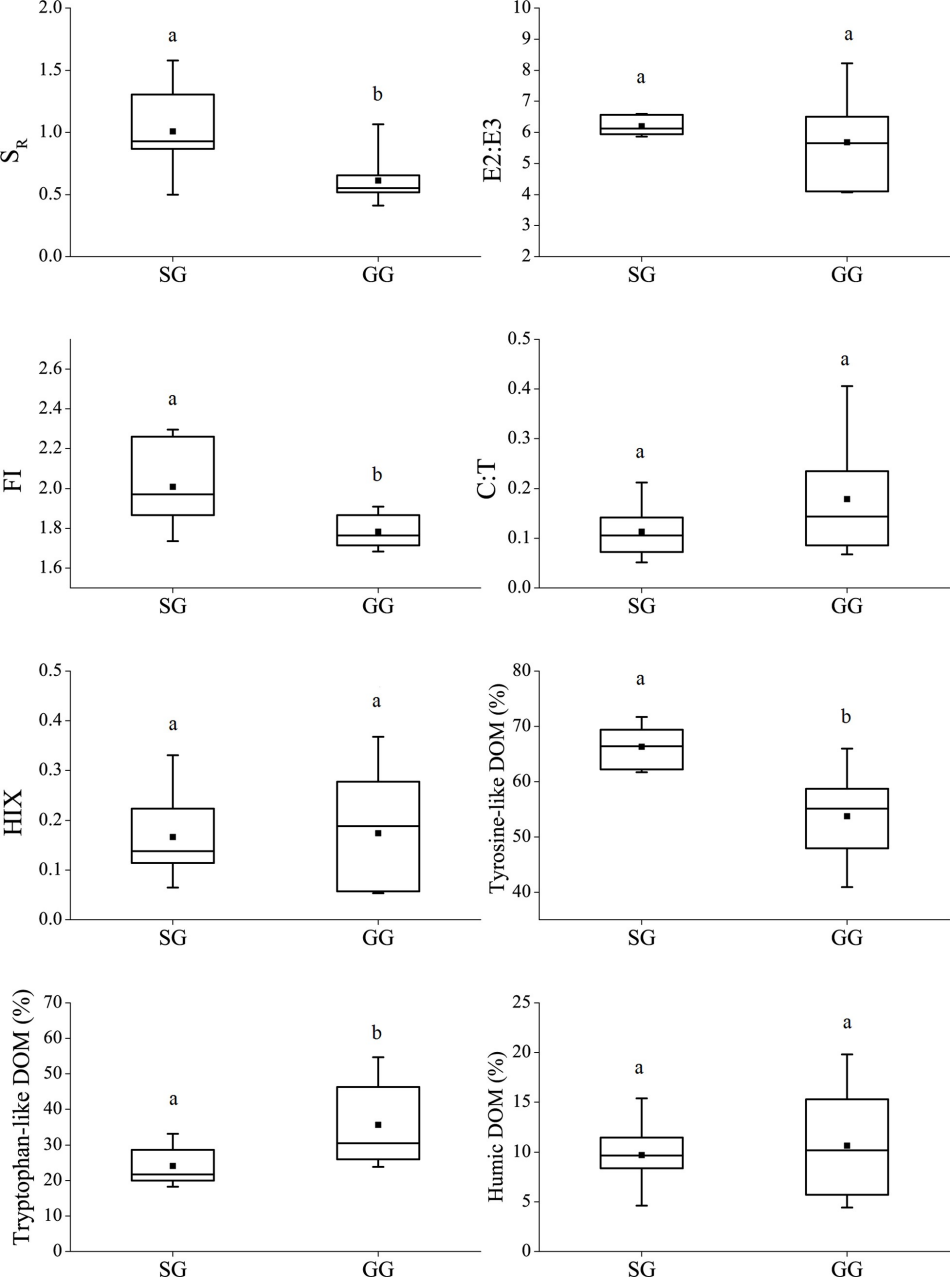
Boxplots of DOM source-composition indices (SR, FI and HIX) and percentage of tryptophan-like compound in leachable DOM in root of SG and GG (N = 8). For each panel, the different lowercase letters denote significant difference between SG and GG (*P* < 0.05). Boxplots show medians (line), means (dot), 1^st^ and 3^rd^ quartiles (box, interquartile range or *IQR*), upper and lower extremes (whiskers). The whiskers were determined as equal to or less extreme than 1.5 times *IQR* against 1^st^ and 3^rd^ quartiles, respectively. The definitions of indices and compounds were defined in the *Method*s section.

Both S_R_ and FI values were significantly higher for SG than GG samples (Fig 3), indicating a lower molecular weight and structural complexity of DOM from SG than from GG. The mean values in C:T and HIX were lower and the mean value in E2: E3 was higher for SG than GG, but no statistically significant differences were detected for these indices (Fig 3). The percent tyrosine-like DOM was significantly higher and the percent tryptophan-like DOM was lower in SG than GG, however, percent humic-like DOM little differed between SG and GG (Fig 3).

## Discussion

### N fertilization enhanced SOC and TN concentrations in SG and GG

Based on our results, we rejected the first hypothesis that the fertilization and crop type interactively influenced SOC and TN. However, we found that N fertilization significantly increased SOC and TN in both SG and GG croplands. This was likely due to the minimal management and mechanical disturbance in our plots, which minimized soil decomposition due to less exposure of below surface soil to air, consequently diminished soil C and N losses in favor of soil C and N accumulations in perennial bioenergy feedstock grasslands [56–58]. In other bioenergy croplands with more common practices, i.e., tillage, plowing and mechanical movement as implemented in conventional croplands (e.g., wheat and corn), more pronounced soil C and N losses may occur due to greater soil decomposition and likely reverse the net SOC and TN gains to net losses. Furthermore, N fertilization could significantly depress soil respiration, microbial biomass and extracellular oxidases’ activities [59, 60], which led to slow turnover of soil C and N cycles and overall SOC and TN accretions [59].

The fertilizer-elevated aboveground biomass yield and the belowground rhizodeposits may also have contributed to the SOC and TN sequestrations by supplying additional amounts of C and N to the soil [7, 56, 61]. This interpretation is supported by the fertilization-enhanced aboveground plant biomass in SG (10–15%) and GG (52–61%) as measured in the same year (e.g., 2015) in this study. Though the belowground root biomass was not qualified in the current fertilization experiment due to technical difficulty (e.g., more than 100cm deep soil excavation and much wider horizontal digging), our literature review showed that bioenergy crop root biomass were not responsive to N fertilization [23, 62]. Despite the less response of total root biomass, both SG and GG possessed a significant volume and mass of coarse root and recalcitrant root exudate to soil [7, 63–65], which had longer residence times in soil in favor of soil C sequestration [66].

### N fertilization effects on SOC and TN depend on fertilization rate

Results from this study supported our second hypothesis that relative to no fertilizer input, fertilization resulted in substantial SOC and TN enrichments only at the relatively high N application rates (168 kg N ha^-1^ yr^-1^) and less likely so at the low fertilization rates (84 kg N ha^-1^ yr^-1^). This finding contrasts with other studies that have demonstrated negative effects of relatively high fertilization rates on soil C and N storage [7, 67]. These negative effects were interpreted as a result of high fertilizer input causing more abundant soil bacteria and low fertilizer use efficiency, which elevated the C and N losses from the soil system [67]. Stewart, Follett [7] documented significant soil C and N accretions even when amended with a relatively low fertilizer input rate similar to this study. These accretions were likely driven by a greater return of aboveground and belowground plant materials to soil after harvesting. Collectively, a threshold fertilizer input rate may exist in our research plots as to their effects on soil C and N storage, but precautions should be taken when different soil and plant types need to be accounted for. Nevertheless, a wide spectrum of fertilization intensity of up to 300 kg N ha^-1^ has been reported in published studies [68, 69], future studies should examine the N fertilization effects in a wide range of fertilization input rates. From a pragmatic perspective, given the pressing need for minimizing the adverse impacts of agriculture on environment, a recommendation is to adopt a sustainable agricultural practice and the important measure is to lower the use of N and other fertilizers [70]. Therefore, it is imperative to elucidate whether a lower-end threshold of fertilizer input exists so that the fertilizer use would continuously benefit both crop productivity and soil fertility with less adverse impact on environment.

### SOC and TN storage correlate with different plant traits

Our analysis of leachable DOM from root supported the third hypothesis that GG root contained higher molecular weight and more structurally complex compound than SG root. This result indicates that GG root would be less readily decomposed compared to SG root. SG is known to have a lower specific root length (i.e., root length per unit root biomass) [27, 71] and GG to have larger coarse root biomass and volume [26]. Given the contrasting root chemistry and morphology between SG and GG, one expected to see a relatively short turnover time for SG root and much longer turnover time for GG root [34]. The slow turnover GG root favored long-term SOC and TN sequestrations [34, 72, 73], likely due to more root-derived organic matter in mineral-associated soil fractions [74] and thus offering explanation of the greater SOC and TN stocks in GG than SG as observed in this study. The assumption is that the contrasting root characteristics observed in the mesocosm experiment will remain under the field fertilization experiment. This is likely true because of similar soil pH in the mesocosm and field experiments, which play a key role in root growth and development [75].

On the other hand, stronger linear relationships of SOC and TN with aboveground plant biomass was identified for SG and less so for GG. Given the significantly greater aboveground plant biomass of SG than GG, these results indicate that the contributions of aboveground plant biomass to belowground soil C and N stocks via litterfall input and turnover were stronger in SG than GG. Considering the aforementioned relationship of GG root with soil C and N storage, our results revealed that the plant traits that contributed to the soil C and N sequestrations varied with bioenergy crop species. It was the aboveground plant biomass of SG and the root of GG that have showed likely associations with their respective soil C and N sequestrations. Despite the long known beneficial role of bioenergy crops on soil C, this study highlighted the need to further elucidate the role of different plant traits (e.g., aboveground vs. belowground) in regulating soil C and N sequestration [76].

## Conclusions

This study demonstrated that relative to no fertilizer input, intensive N fertilization (e.g., HN) could significantly increase SOC and TN in bioenergy cropland surface soils (0-15cm). Meanwhile, GG showed significantly higher SOC and TN and significantly lower aboveground biomass than SG. There were strong positive linear relationships of SOC and TN with aboveground biomass in SG, and structurally more complex and less readily decomposed root DOM in GG. This suggested that the intensive N fertilization induced C and N accumulations in soil may be more likely mediated by the aboveground biomass in SG and root chemistry and morphology in GG. Future studies should examine the root characteristics in different bioenergy croplands under the field fertilization experiment.

## Supporting information

S1 Table Characteristics of the three fluorescence components identified by PARAFAC model and their attributed sources. The modeling method was described in *Method* section.
(DOCX)

S1 Data Dataset of SOC, TN and C: N, plant aboveground (ABG) biomass, and root DOM chemistry. (XLSX)
