# Supplementary material for: Effects of nitrogen fertilization and bioenergy crop type on topsoil organic carbon and total Nitrogen contents in middle Tennessee USA
Source: PLoS One. 2020 Mar 30;15(3):e0230688. doi: 10.1371/journal.pone.0230688 (PMC7105129; doi:10.1371/journal.pone.0230688)
Supplement: S1 Table — The modeling method was described in Method section. (DOCX) [file pone.0230688.s001.docx]

**Supplementary Material**

**Effects of Nitrogen Fertilization and Bioenergy Crop Type on Topsoil Organic Carbon and Total Nitrogen Contents in Middle Tennessee USA**

Jianwei Li^1*^, Siyang Jian^1^, Chad S. Lane^2^, YueHan Lu^3^, Xiaorui He^3^, Gangsheng Wang^4^, Melanie A. Mayes^5^, Kudjo E Dzantor^1^, Dafeng Hui^6^

^1^Department of Agricultural and Environmental Sciences, Tennessee State University, Nashville, TN 37209

^2^Department of Earth and Ocean Sciences, University of North Carolina Wilmington, Wilmington, NC 28403

^3^Department of Geological Sciences, University of Alabama, Tuscaloosa, Alabama 35487, USA

^4^Institute for Environmental Genomics and Department of Microbiology & Plant Biology, University of Oklahoma, Norman, Oklahoma, 73019, USA

^5^Climate Change Science Institute and Environmental Sciences Division, Oak Ridge National Laboratory, Oak Ridge, Tennessee 37831, USA

^6^Department of Biological Sciences, Tennessee State University, Nashville TN 37209

* Corresponding author

Jianwei Li

Department of Agriculture and Environmental Sciences

Tennessee State University

Nashville, TN 37209, USA

Phone: (615) 963-1523

Fax: (615) 963-5436

Table S1. Characteristics of the three fluorescence components identified by PARAFAC model and their attributed sources. The modeling method was described in *Method* section.

([Wheeler et al. 2017](#_ENREF_5)) ([Cory and McKnight 2005](#_ENREF_2)) ([Lu et al. 2015](#_ENREF_4)) ([Coble 2007](#_ENREF_1)) ([Fellman et al. 2010](#_ENREF_3))

References

Coble, P. G. 2007. Marine optical biogeochemistry: The chemistry of ocean color. Chemical Reviews **107**:402-418.

Cory, R. M., and D. M. McKnight. 2005. Fluorescence spectroscopy reveals ubiquitous presence of oxidized and reduced quinones in dissolved organic matter. Environmental science & technology **39**:8142-8149.

Fellman, J. B., E. Hood, and R. G. M. Spencer. 2010. Fluorescence spectroscopy opens new windows into dissolved organic matter dynamics in freshwater ecosystems: A review. Limnology and Oceanography **55**:2452-2462.

Lu, Y. H., J. W. Edmonds, Y. Yamashita, B. Zhou, A. Jaegge, and M. Baxley. 2015. Spatial variation in the origin and reactivity of dissolved organic matter in Oregon-Washington coastal waters. Ocean Dynamics **65**:17-32.

Wheeler, K. I., D. F. Levia, and J. E. Hudson. 2017. Tracking senescence-induced patterns in leaf litter leachate using parallel factor analysis (PARAFAC) modeling and self-organizing maps. Journal of Geophysical Research: Biogeosciences **122**:2233-2250.
